# Community participation and rural residents’ sense of gain, happiness, and security: the mediating role of collective psychological ownership

**DOI:** 10.3389/fpsyg.2026.1764190

**Published:** 2026-08-07

**Authors:** Jianchun Yang, Mengya Qi, Yan Kou, Yu Han

**Affiliations:** 1 School of Business Administration, Guizhou University of Finance and Economics, Guiyang, Guizhou, China; 2 School of Digital Economy and Finance, Guizhou University of Commerce, Guiyang, Guizhou, China

**Keywords:** Community participation, collective psychological ownership, sense of gain, happiness, sense of security, government trust

## Abstract

Enhancing rural residents’ sense of gain, happiness, and security is central to China’s rural revitalization strategy and to residents’ aspirations for a better life. Yet in rural tourism destinations, the pathway through which community participation is translated into residents’ perceived quality of life remains insufficiently clarified. This study examines the relationships between community participation and residents’ sense of gain, happiness, and security, and further tests the mediating role of collective psychological ownership and the moderating role of government trust. Using 536 valid questionnaires collected from five rural tourism villages in Guizhou Province, China, the study analyzes the data with SPSS 22.0 and SmartPLS 3.0. The results indicate that (1) community participation is positively associated with residents’ sense of gain, happiness, and security; (2) collective psychological ownership mediates these relationships; (3) government trust strengthens the positive association between community participation and collective psychological ownership. These findings enriches the theoretical perspective on community participation and provides practical insights into improving rural residents’ sense of gain, happiness, and security.

## 1 Introduction

In recent years, growing attention to sustainable development, inclusive growth, and rural revitalization has made residents’ quality of life and subjective well-being an important academic and policy concern. In China, rural revitalization and common prosperity have further heightened this issue. The 19th and 20th National Congresses of the Communist Party of China emphasized that people’s sense of gain, happiness, and security should become more substantial, more secure, and more sustainable, and the 2025 Government Work Report again called for continuously enhancing these three dimensions. These policy commitments suggest that the sense of gain, happiness, and security are not merely subjective feelings but important indicators for evaluating rural development and livelihood improvement. As a pathway for rural industrial revitalization and inclusive development, rural tourism provides a critical context in which livelihoods, development opportunities, and subjective well-being may be reshaped. Therefore, exploring how rural tourism can enhance residents’ sense of gain, happiness, and security, so as to effectively improve people’s well-being and continuously fulfill their aspirations for a better life, remains an important issue at present and in the future.

The sense of gain, happiness, and security reflect residents’ subjective evaluations of development outcomes, living conditions, and the stability of the external environment (Duan and Wu, 2021). In rural tourism destinations, residents are not merely recipients of tourism impacts but key actors in local development. Community participation, defined here as residents’ sustained involvement in tourism planning, development, operation, management, and supervision, thus provides an analytical lens for understanding how rural tourism shapes residents’ subjective perceptions. Although previous studies have linked community participation to fairness perception, sense of belonging, and conflict perception (Ma, 2015; Lin et al., 2019; Wang et al., 2021), its relationship with residents’ sense of gain, happiness, and security as integrated indicators of perceived quality of life remains underexplored. This study therefore examines whether and how community participation predicts these three perceptions among rural tourism residents.

To explain this relationship, the study introduces collective psychological ownership as a mediating variable. Rural tourism development is not simply an individual economic activity but a collective process involving community resource use, public deliberation, benefit distribution, and shared maintenance. Through this process, residents may obtain tangible benefits while developing a shared sense of possession, responsibility, and belonging toward the community (Huo et al., 2023), which may in turn shape their sense of gain, happiness, and security. The study also examines government trust as a boundary condition because community participation occurs within an institutional context. In rural tourism destinations, government plays an important role in resource integration, institutional provision, and development guidance; residents’ trust in government may therefore influence how they interpret participation and the extent to which participation is transformed into collective psychological ownership (Zheng et al., 2022).

This study contributes to the literature in three ways. First, by comprehensively examining residents’ sense of gain, happiness, and sense of security, this research moves beyond prior studies that typically focused on a single dimension, thereby offering a more holistic analytical framework. Second, by introducing collective psychological ownership as a mediating variable, the study provides a novel theoretical lens for explaining how community participation predicts residents’ senses of gain, happiness, and security in rural tourism contexts. Finally, the incorporation of government trust as a moderator reveals the boundary mechanism of community participation’s influence on collective psychological ownership. This not only enriches the research on government trust but also deepens the theoretical understanding of how institutional factors shape residents’ perceptions and psychological states in rural tourism development.

## 2 Literature review and research hypothesis

### 2.1 Community participation

Community participation refers to the extent to which members are involved in the daily affairs of their community (Lee, 2013). In tourism, it is a multidimensional construct spanning decision-making, planning, development, management, and supervision (Guo et al., 2015). Rather than treating communities merely as beneficiaries, community participation positions residents as key actors in tourism development, enabling local opinions and needs to be incorporated into decision-making and promoting both tourism sustainability and collective community prosperity. Community participation also involves cooperation among community members, including the allocation of material resources, knowledge sharing, learning, and transformation. Its aim is to achieve shared objectives, improve local communities, and promote individual well-being (McCloskey et al., 2011). In community-based tourism, residents’ engagement helps align development with local expectations and supports both economic growth and quality of life (Wang et al., 2021). Existing tourism research has examined community participation in relation to rural tourism, sustainable development, and community empowerment, including participation models, mechanisms, resident perceptions, motivations, and benefit distribution (e.g., Liu et al., 2025; Pang et al., 2024; Witchayakawin et al., 2024). However, few studies have systematically analyzed the intrinsic connection between community participation and residents’ sense of gain, happiness, and security from the perspective of rural tourism area residents.

### 2.2 Sense of gain, happiness, sense of security

Meeting people’s aspirations for a better life is a central task of social development at the present stage. As key indicators of this pursuit, the sense of gain, happiness, and security have attracted increasing academic attention, as they reflect the extent to which individuals perceive their aspirations for a better life as being fulfilled. Sense of gain refers to the positive psychological state experienced as circumstances improve and social status rises, emphasizing perceived improvements across material, spiritual, and rights-related dimensions (Gu et al., 2020). Happiness refers to a subjective psychological experience formed through the comparison between life expectations and actual living conditions, reflecting the degree of alignment between one’s envisioned ideal life and lived reality (Diener et al., 1985). Sense of security refers to the emotional experience that arises when external environments and conditions satisfy an individual’s security needs, encompassing subjective evaluations of environmental stability, trust in the social environment, and confidence in interpersonal relationships (Zou and Meng, 2019).

These three dimensions are interdependent and mutually reinforcing: sense of gain underpins both happiness and security, happiness reflects the realization of gain and security, and security provides a safeguard for both. In the broader context of comprehensive rural revitalization, all three have been recognized as key indicators for evaluating residents’ quality of life. Although they together provide a multidimensional view of well-being, each emphasizes a distinct aspect of residents’ lived experience. An integrative examination of these three dimensions is therefore necessary.

Yet a review of existing studies suggests that these three constructs have tended to be examined separately rather than as interrelated dimensions. Studies on sense of gain have emphasized objective attainment, institutional governance, and process participation, with discussions centering on income growth, livelihood improvement, government performance, public service provision, and political participation (e.g., Zheng, 2020; Tang and Su, 2022; Huang et al., 2022). Research on happiness has largely examined how tourism development, changes in living conditions, and residents’ perceptions of tourism-related benefits and costs shape well-being (Li and Sun, 2025; Uysal et al., 2025). Studies on sense of security have focused on destination safety perception and risk perception, highlighting safety information, environmental and service protection, governance capacity, and the distinction between expected and experienced safety (e.g., Zou and Meng, 2019; Zou and Yu, 2022). Taken together, existing studies still tend to treat these three constructs along separate lines, and few have incorporated them into a unified analytical framework. Research that further links all three constructs with community participation is even scarcer. Grounded in the context of comprehensive rural revitalization, the present study addresses this gap by investigating how community participation predicts rural tourism residents’ sense of gain, happiness, and security.

### 2.3 Community participation and residents’ sense of gain, happiness, and security

Existing empirical research has linked sense of gain to political participation and public service engagement. Tang and Su (2022) indicate that both institutional and non-institutional participation influence public sense of gain, although their effects differ. Zheng et al. (2022) further found that individual participation in public services enhances sense of gain only under conditions of high government transparency. These findings suggest that community participation may contribute positively to residents’ perceived sense of gain. This relationship is particularly relevant in rural tourism destinations, where residents are both direct beneficiaries of development and endogenous actors in the sustainable growth of rural tourism. Active participation enables residents to access employment opportunities and income streams generated by tourism development, providing important economic support for households that previously lacked stable income sources (Spencer and Nsiah, 2013). Participation in community tourism management and decision-making also allows residents to express interests, negotiate issues, and engage in community governance, thereby strengthening their perceptions of developmental rights and entitlement to share in development outcomes (Niu et al., 2020). On this basis, the following hypothesis is proposed:

*H1a*: Community participation is positively associated with the sense of gain of residents in rural tourism destinations.

Existing research has established a strong association between community participation and well-being. Engagement in sports, leisure activities, and other community initiatives can enhance individual happiness (Ahn, 2020), while community participation can foster residents’ perceptions of value and fairness, thereby contributing to greater well-being (Lv and Xie, 2017). Chen and Zhang (2022) further indicate that community involvement enhances well-being among older adults by strengthening community identity. These findings suggest that community participation may also contribute to residents’ happiness in rural tourism destinations. In community tourism development, residents are not merely passive recipients of change but co-constructors of community development. Through participation, they may develop a stronger sense of self-worth, accomplishment, and positive self-concept, which can lead them to evaluate their current lives more positively and perceive their living conditions as closer to their ideal life expectations (Chen and Zhang, 2022). On this basis, the following hypothesis is proposed:

*H1b*: Community participation is positively associated with the happiness of residents in rural tourism destinations.

Sense of security is important to both the individual and collective well-being of residents. Prior research indicates that community structure, including sociodemographic characteristics and homeownership rates, may affect residents’ sense of security; for example, perceived insecurity often increases with age (Collins and Guidry, 2018). Beyond structural factors, participation and social capital may also shape residents’ sense of security. In community tourism, participation enables residents to better understand community rules, tourism development arrangements, and related environmental changes, thereby strengthening their understanding of the community’s operational logic and future direction (Collins et al., 2014). Improvements in infrastructure, public services, and order maintenance associated with tourism development may further reinforce residents’ perceptions of environmental stability and reliable community operations. Sustained participation can also strengthen information exchange, relational ties, and support networks, making residents more aware that they belong to a mutually supportive collective. As a result, they are more likely to perceive their community as stable, orderly, and dependable. On this basis, the following hypothesis is proposed:

*H1c*: Community participation is positively associated with the sense of security of residents in rural tourism destinations.

### 2.4 Mediating role of collective psychological ownership

Pierce et al. (2001) define psychological ownership as an individual-level state in which a person feels that a target, or part of it, is “mine”. Pierce and Jussila (2010) extend this concept to the group level, where collective psychological ownership refers to a shared consciousness that a target belongs to “us” as a whole. Although individual and collective psychological ownership both center on possession, they differ in their subject and formation. Individual psychological ownership arises from the interaction between an individual and a target and is shaped by personal control, knowledge, and investment. By contrast, collective psychological ownership emerges from dynamic interactions among group members, is influenced by collectivist values, task interdependence, and team cohesion, and requires a shift in the reference point of ownership from the “self” to the “group.” Thus, it is not merely an aggregation of individual ownership feelings, but a group-level psychological state formed through shared experience, mutual perception, and group integration.

Community participation in rural tourism is not merely isolated individual action but involves residents’ joint participation, collective deliberation, and shared governance in community planning, resource use, public decision-making, benefit distribution, and social order maintenance. In this context, the object of ownership is the community as a whole, whose development is jointly shaped, maintained, and shared by residents. Collective psychological ownership is therefore better suited than individual psychological ownership to capture the communal bond generated through participation and its influence on residents’ subjective perceptions. Although recent scholarship has examined the antecedents, consequences, measurement, mechanisms, and mediating or moderating effects of collective psychological ownership (Giordano et al., 2020; Su and Ng, 2019), few studies have applied it to tourism research or linked it specifically to community participation. This study therefore investigates how collective psychological ownership mediates the relationship between community participation and residents’ sense of gain, happiness, and security.

According to psychological ownership theory, psychological ownership is driven by efficacy, self-identity, and spatial needs, and develops through control, intimate familiarity, and self-investment (Avey et al., 2009). In community tourism, residents’ participation can foster collective psychological ownership through these pathways. First, participation enables shared control over tourism development by granting residents involvement and information rights, strengthening their collective voice, status, identity, and sense of control (Liu et al., 2021). Second, participation deepens shared familiarity with the community through decision-making, management, supervision, collaboration, information exchange, and knowledge sharing. Third, participation enhances shared investment, as residents with participatory and informational rights are more likely to devote effort and commitment to supporting the community as a whole. Together, these processes foster collective psychological ownership.

Psychological ownership signals to residents that the community belongs to them and that they are part of it. When residents develop collective psychological ownership, they are more likely to form emotional attachments, strengthen intrinsic motivation, and view community development outcomes as personally meaningful. According to endowment effect theory, individuals assign higher value to possessions or resources they perceive as their own (Tang et al., 2024). Thus, in community tourism, residents with collective psychological ownership may attach greater value to the community and perceive its development outcomes as more tangible and significant. They may also regard the loss of emotional attachment as a meaningful deprivation. When residents feel that the community belongs to “us,” they are more likely to cherish its development outcomes and experience a stronger sense of gain (Dugstad et al., 2024). In this way, collective psychological ownership bolsters residents’ perceived value of the community and contributes to their sense of gain.

Organizational behavior research has established a positive link between psychological ownership and well-being. Fan et al. (2019) examined how psychological ownership affects employee well-being through job involvement, while Ali et al. (2021) found that psychological ownership mediates the relationship between managerial guidance and workplace well-being. Building on this literature, this study posits that collective psychological ownership is positively associated with residents’ happiness. Ryan and Deci (2000) argue that individuals experience greater well-being when social environments and interpersonal factors support the realization of their potential, capabilities, and emotional needs. In community tourism, collective psychological ownership can strengthen residents’ bonds with the community and foster psychological closeness and responsibility. It may also encourage continuous investment in the community, satisfying residents’ need for competence and further promoting happiness.

Sense of security reflects individuals’ trust in the stability and reliability of the social environment and in the mutual trust embedded in social relationships. When residents develop collective psychological ownership, they are more likely to feel that the community belongs to them and to assume responsibility for its safety and development (Kaur et al., 2013). This shared ownership can motivate more active participation in community affairs, deepening residents’ understanding of and influence over community operations and future development, thereby strengthening their confidence in what lies ahead (Li et al., 2012). Collective psychological ownership may also reinforce cooperation and mutual support. When residents recognize that they share ownership of the community, they are more likely to see themselves as part of a stable and mutually supportive collective. This shared ownership can function as a psychological defense mechanism, helping residents feel safer when facing external uncertainties or challenges.

Drawing upon this, the subsequent hypotheses are posited:

*H2a*: Collective psychological ownership mediates the interrelation between community participation and residents' sense of gain;

*H2b*: Collective psychological ownership mediates the interrelation between community participation and residents' happiness;

*H2c*: Collective psychological ownership mediates the interrelation between community participation and residents' sense of security.

### 2.5 Moderating role of government trust

Government trust refers to the public’s psychological state and attitude toward government in terms of expectations and perceptions (Fan et al., 2022). In community tourism development, trust between government and residents may shape the trajectory of community development. Prior research shows that trust in local government is closely related to tourism development and serves as an important precondition for residents to understand tourism governance arrangements (Nunkoo, 2015). It also influences residents’ behavior in community tourism participation (Jia et al., 2021). Higher government trust can enhance residents’ recognition of governance rules, development directions, and policy implementation, while reducing concerns about ineffective participation, benefit loss, and procedural unfairness. This may lead residents to interpret community participation as a process of jointly building and maintaining the community (Nunkoo, 2015; Ouyang et al., 2017). However, excessively high government trust may also produce a countervailing effect. When residents view community development as primarily government-led and government-responsible, their sense of agency and shared responsibility may weaken, and participation may become compliance with government arrangements rather than proactive community-building, potentially fostering dependency or free-rider behavior (Ouattara and Steenvoorden, 2024; Cui and Li, 2025). Thus, government trust may function as a conditional factor that can both motivate and inhibit residents’ actions, rather than as a uniformly positive participation resource. Its moderating role in the relationship between community participation and collective psychological ownership therefore warrants further investigation.

In rural tourism destinations in ethnic minority areas of Southwest China, tourism development is typically characterized by strong government embeddedness, with local governments playing key roles in policy formulation, development planning, resource integration, and institutional provision. At the same time, such development relies on community empowerment, public participation, and residents’ stewardship of local cultural resources. Although governments perform important organizational and coordinating functions, they cannot substitute for residents’ role in community construction (Guo et al., 2024; Chen et al., 2017; Wang et al., 2020). In this context, government trust does not replace residents’ participation; rather, it shapes how residents interpret their involvement and thereby influences the transformation from community participation to collective psychological ownership.

Social identity theory argues that individuals define their self-concept through identification with social groups or organizations and gradually establish social identity through cognitive investment and self-classification (Lee et al., 2016). From this perspective, residents with high government trust are more likely to participate actively in community tourism affairs, support government decisions, believe that government will respond to their needs and protect their interests, and identify with the collective goal of tourism development. This positive perception can help transform community participation into collective psychological ownership. Conversely, when government trust is low, residents may question policy intentions and governance fairness, viewing participation as a passive obligation rather than co-construction, thereby weakening the formation of collective psychological ownership. On this basis, the following hypothesis is proposed:

*H3*: Government trust plays a positive moderating role between community participation and collective psychological ownership.

**Figure 1:**
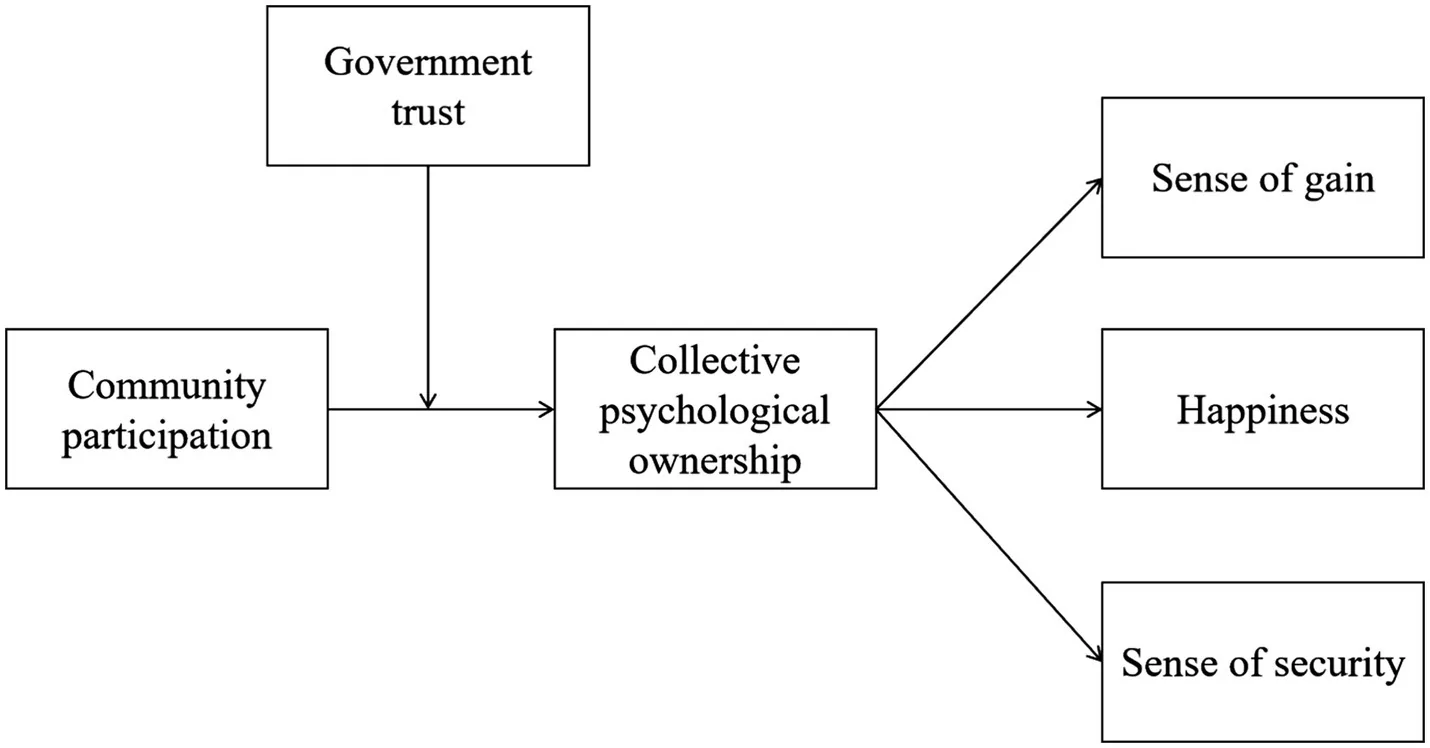
Conceptual model.

The conceptual model is illustrated in Figure 1.

## 3 Methodology

### 3.1 Research methods

This study first conducted descriptive statistical analysis using SPSS 22.0 and then tested the model with SmartPLS. PLS-SEM was selected for three reasons. First, PLS can estimate all hypothesized relationships simultaneously, including models with multiple dependent variables, and is robust when data deviate from multivariate normality or sample sizes are relatively small (Chin et al., 2003). This is appropriate for questionnaire data from rural tourism residents, given respondents’ heterogeneity in educational attainment, income level, and life background. Second, the proposed model involves relatively complex relationships among variables and aims to explore a new model rather than verify an established one. Third, this study focuses on path relationships and predictive power rather than the overall goodness-of-fit of a covariance-based structural model. Thus, the variance-based and prediction-oriented PLS-SEM approach is suitable.

### 3.2 Construct and measurement

To ensure the reliability and validity of the measurement instruments, all items were developed based on existing literature. Scales derived from prior international studies were translated from English into Chinese by a bilingual translator and then back-translated into English by a second bilingual translator. The back-translated version was compared with the original scale, and items with discrepancies were revised and back-translated again to avoid translation bias. Village cadres from five representative rural tourism destinations were then invited to complete a pilot version of the questionnaire. Based on their feedback and the pilot survey results, the items were revised to ensure clear wording, local comprehensibility, and consistency with local expressions. The final questionnaire used a five-point Likert scale ranging from 1 (“strongly disagree”) to 5 (“strongly agree”).

Community participation was measured using a four-item scale developed by Rasoolimanesh et al. (2017) and Tosun (2000). Collective psychological ownership was assessed with a four-item scale adapted from Liu et al. (2021). Sense of gain was measured with a nine-item scale developed by Zheng (2020), happiness with a five-item scale developed by Diener et al. (1985), sense of security with an eight-item scale developed by Zheng (2020), and government trust with a four-item scale developed by Jia et al. (2021). Demographic variables included gender, age, educational attainment, and monthly income level.

### 3.3 Sample and data collection

To ensure sample representativeness, questionnaires were distributed in five rural tourism villages in Guizhou Province: Nanhua Miao Village, Guiding Yin Village, Langde Shangzhai, Zhaoxing Dong Village, and Xijiang Miao Village. These sites were selected for two reasons. First, Guizhou is a major tourism province in China with a relatively high level of tourism development, making it suitable for collecting tourism-related samples. Second, the five villages represent concentrated areas of tourism development and different stages of the tourism destination life cycle. Specifically, Nanhua Miao Village is in the exploration stage; Langde Shangzhai and Guiding Yin Village are in the development stage; and Zhaoxing Dong Village and Xijiang Miao Village are in the maturity stage. These sites therefore show typicality and representativeness. In addition, local villagers have relatively high levels of tourism participation and direct knowledge of the tourism sector, which supports their comprehension of the questionnaire and enhances the credibility of the survey data.

The survey was conducted in two stages. A preliminary survey was carried out in Xijiang Miao Village in March 2024, and the results indicated good questionnaire quality, requiring only minor clarifications. The formal survey was conducted on site in May 2024. Trained researchers, assisted by local students, randomly visited households in Xijiang Miao Village, Langde Shangzhai, Nanhua Miao Village, Zhaoxing Dong Village, and Guiding Yin Village. To avoid overconcentration of samples in larger villages and ensure adequate representation of each case village, a balanced sampling allocation was adopted across the five ethnic villages. A total of 650 questionnaires were distributed, with 130 allocated to each village. Of these, 560 were returned, including 110 from Nanhua Miao Village, 110 from Langde Shangzhai, 112 from Guiding Yin Village, 113 from Zhaoxing Dong Village, and 115 from Xijiang Miao Village. After data screening, 536 valid questionnaires were retained, including 106 from Nanhua Miao Village, 106 from Langde Shangzhai, 107 from Guiding Yin Village, 108 from Zhaoxing Dong Village, and 109 from Xijiang Miao Village, yielding an effective response rate of 82.46%.

The sample was predominantly female (67.9%). The largest age groups were 50–65 years (35.4%) and 34–49 years (25.6%), followed by 18–33 years (20.9%), with fewer respondents aged 66 and above or under 18. In terms of education, most respondents had junior high school education (48.1%), followed by high school or vocational high school (34.7%) and primary school or below (14%), while undergraduate or junior college respondents accounted for a smaller proportion. Regarding monthly per capita income, the largest group earned 1,500–2,500 yuan (37.6%), followed by those earning less than 1,500 yuan (28.2%). For length of residence, respondents living in the area for 11–20 years accounted for the largest share (29.3%), followed by 21–30 years (24.6%), while those with more than 40 years of residence accounted for the smallest share (7.8%).

### 3.4 Common method bias

Harman’s single-factor test was conducted to examine potential common method bias. The results show that seven factors had eigenvalues greater than 1 and together explained 72.95% of the variance. The first factor explained 39.64% of the total variance, slightly below the 40% threshold. Therefore, common method bias is unlikely to pose a serious concern in this study (Podsakoff and Organ, 1986).

## 4 Analysis and results

### 4.1 Assessing the reliability and validity of the measures

SmartPLS 3.0 was used to assess the reliability and validity of the constructs. Following the criteria proposed by Hair et al. (2017), all constructs showed Cronbach’s alpha values above 0.70, and composite reliability (CR) values exceeded 0.70, indicating satisfactory reliability. As shown in Table 1, the measurement scales met the recommended reliability thresholds. Furthermore, all constructs reported average variance extracted (AVE) values greater than 0.50, confirming satisfactory convergent validity. Following Fornell and Larcker (1981), the square roots of the AVE values were compared with the inter-construct correlation coefficients. As shown in Table 2, the diagonal AVE square roots exceeded the correlations among constructs, supporting discriminant validity.

**Table 1:** Test of measurement model.

Latent variables	Items	Factor loading	Cronbach’s Alpha	CR	AVE
Community participation	I participated in the daily management of rural tourism development (tourism development meetings, tourism development education and training, ecological environmental protection, etc.)	0.934	0.926	0.947	0.818
	I participated in the benefit distribution of rural tourism development (sharing the benefits brought by tourism development with tourism enterprises and investors)	0.892			
	I participated in the decision-making of rural tourism development (scenic spot construction, tourism planning, etc.)	0.891			
	I participated in the reception activities of rural tourism (performing artistic shows for tourists, preparing B&Bs, catering, etc.)	0.901			
Collective psychological ownership	We (the residents of the rural tourism destination and I) collectively feel that this community is our community	0.915	0.890	0.924	0.753
	We (the residents of the rural tourism destination and I) all feel a very high degree of collective ownership for this community	0.845			
	We (the residents of the rural tourism destination and I) collectively feel that this community belongs to us together	0.848			
	All the residents in this community feel that we own the community collectively	0.861			
Sense of gain	I can feel the harmonious atmosphere and good trust of this community	0.924	0.945	0.953	0.694
	I can feel our community is developing in the direction of fairness and justice	0.809			
	I can feel my life is getting more dignified	0.832			
	I can feel a sense of accomplishment and pride in participating in the development of our community	0.850			
	Now, I get more development dividends than ever before	0.823			
	At present, I can feel a significant increase in income	0.805			
	At present, I can feel problems such as education, medical care and housing in our community are being gradually solved and improved	0.806			
	I can feel the ecological environment and other conditions in our community are developing in a good direction	0.823			
	I can participate in the development of our community with my own wisdom and strength	0.820			
Happiness	In most ways my life is close to my ideal	0.918	0.908	0.932	0.732
	The conditions of my life are excellent	0.886			
	I am satisfied with my life	0.830			
	So far I have gotten the important things I want in life	0.813			
	If I could live my life over, I would change almost nothing	0.827			
Sense of security	My living environment, traffic situation is safe	0.932	0.942	0.951	0.711
	My life and property safety can be guaranteed	0.844			
	I am very satisfied with the current security situation of our community	0.832			
	I rarely worry about the day-to-day security of our community	0.809			
	I believe that I can improve my status in the community through my own efforts	0.830			
	Our rights will be guaranteed when we participate in the development of our community	0.833			
	It is entirely predictable that our community will develop steadily and have a long-term stability	0.829			
	I believe that my income or that of my family will increase steadily	0.830			
Government trust	Trust in tourism decisions made by local government	0.769	0.818	0.878	0.643
	Trust in local government to look after the interest of this community	0.860			
	Trust in local government to do what is right in tourism	0.812			
	Trust in local government to make a serious effort to incorporate residents into event planning process	0.762			

**Table 2:** Reliabilities, square root of AVEs, and correlations.

Latent variables	CP	CPO	SG	Happiness	SS	GT
Community participation (CP)	0.905					
Collective psychological ownership (CPO)	0.260	0.868				
Sense of gain (SG)	0.390	0.509	0.833			
Happiness	0.508	0.466	0.575	0.856		
Sense of security (SS)	0.309	0.525	0.613	0.491	0.843	
Government trust (GT)	0.157	0.242	0.131	0.185	0.192	0.802

Diagonal elements are the square root of the AVE. The off-diagonal elements are the correlations among latent variables.

### 4.2 Analysis of the structural model

The structural model was evaluated by examining multicollinearity, path coefficients, R^2^ values, effect sizes (f^2^), and predictive relevance (Q^2^), as shown in Tables 3, 4.

**Table 3:** Structural model evaluation results.

Hypotheses	Without control variables		With control variables	
	β	*p*	β	*p*
Community participation → Sense of gain	0.276***	<0.001	0.186***	<0.001
Community participation → Happiness	0.415***	<0.001	0.226***	<0.001
Community participation → Sense of security	0.184***	<0.001	0.103*	<0.05
Community participation → Collective psychological ownership	0.279***	<0.001	0.180***	<0.001
Collective psychological ownership → Sense of gain	0.438***	<0.001	0.410***	<0.001
Collective psychological ownership → Happiness	0.359***	<0.001	0.304***	<0.001
Collective psychological ownership → Sense of security	0.477***	<0.001	0.453***	<0.001
Government trust → Effect of community participation on collective psychology	0.459***	<0.001	0.450***	<0.001

***p < 0.001, *p < 0.05 (based on a two-tailed test).

**Table 4:** Mediation test results.

Hypothesized path	Indirect effect	t-value	95% confidence interval	Test result
Community participation → Collective psychological ownership → Sense of gain	0.074	5.804	[0.033, 0.117]	Supported
Community participation → Collective psychological ownership → Happiness	0.055	5.386	[0.024, 0.089]	Supported
Community participation → Collective psychological ownership → Sense of security	0.081	5.914	[0.038, 0.128]	Supported

Variance inflation factor (VIF) values ranged from 1.035 to 1.198, confirming the absence of multicollinearity and remaining well within the acceptable limit of 5 (O’Brien, 2007).

Using the bootstrap resampling procedure with 5,000 iterations, we estimated model parameters and evaluated the significance of the structural paths. Before the inclusion of control variables, community participation is positively associated with residents’ sense of gain (*β* = 0.276, *p* < 0.001), happiness (*β* = 0.415, *p* < 0.001), and sense of security (*β* = 0.184, *p* < 0.001), thereby providing initial support for H1a, H1b, and H1c. Community participation is also positively related to collective psychological ownership (*β* = 0.279, *p* < 0.001), and collective psychological ownership is positively associated with sense of gain (*β* = 0.438, *p* < 0.001), happiness (*β* = 0.359, *p* < 0.001), and sense of security (*β* = 0.477, *p* < 0.001).

After controlling for age, education, monthly income, length of residence, and village development stage, community participation remains positively associated with sense of gain (*β* = 0.186, *p* < 0.001), happiness (*β* = 0.226, *p* < 0.001), and sense of security (*β* = 0.103, *p* < 0.05), and is also positively related to collective psychological ownership (*β* = 0.180, *p* < 0.001). At the same time, collective psychological ownership remains positively associated with sense of gain (*β* = 0.410, *p* < 0.001), happiness (*β* = 0.304, *p* < 0.001), and sense of security (*β* = 0.453, *p* < 0.001). Compared with the model without control variables, these coefficients become weaker but remain significant, indicating that individual background and contextual factors account for part of the variance.

**Figure 2:**
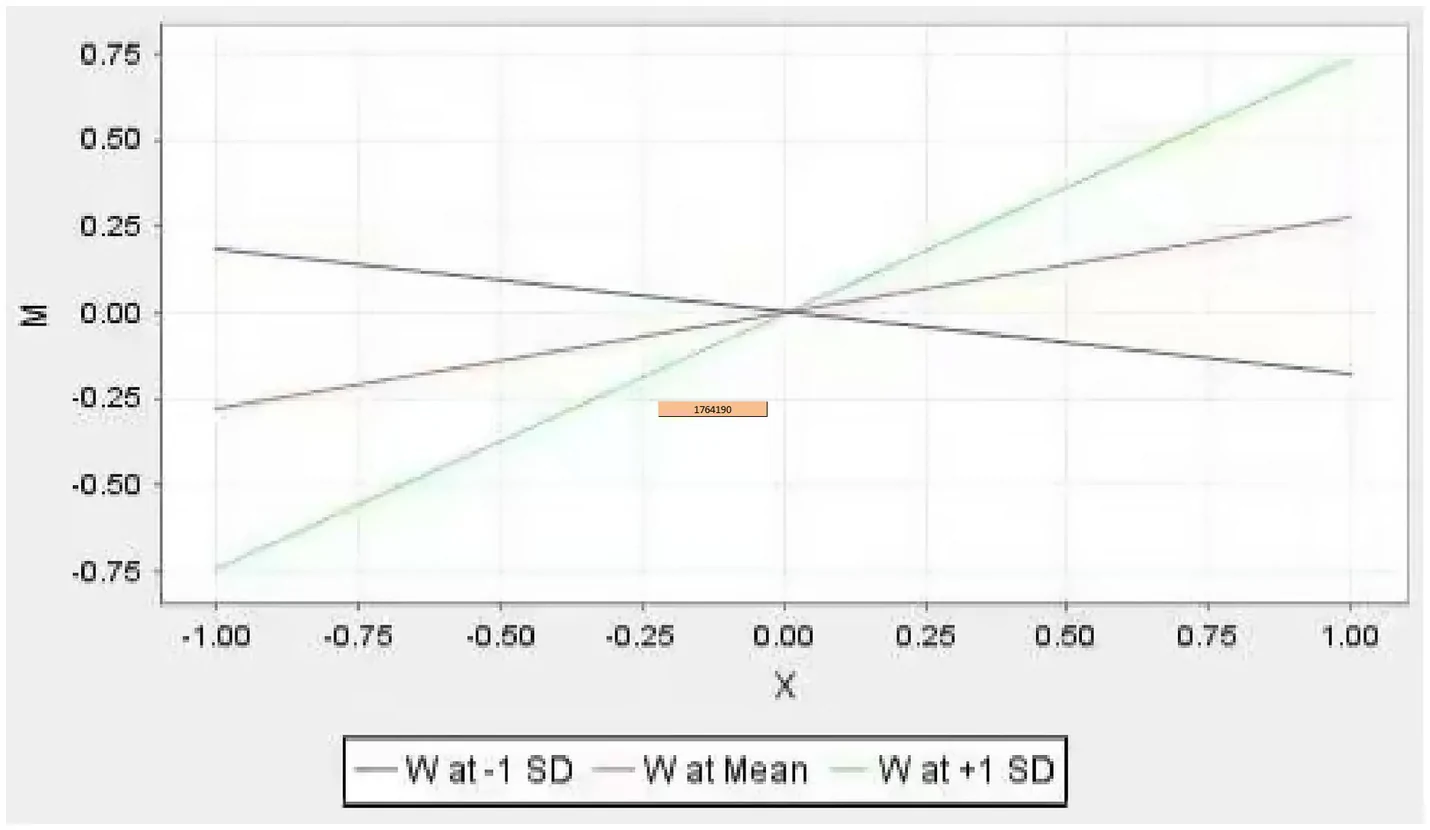
Modulation response curve.

With respect to the moderating role of government trust, community participation and government trust were mean-centered before constructing the interaction term, and significance was tested using 5,000 bootstrap resamples. The results show that the interaction term adds explanatory power to the model (ΔR^2^ = 0.246), and that the interaction between community participation and government trust is positively associated with collective psychological ownership (*β* = 0.450, *p* < 0.001). Figure 2 further indicates that this positive relationship is stronger at higher levels of government trust, thereby supporting H3.

The study further examined the mediating role of collective psychological ownership in the relationships between community participation and residents’ sense of gain, happiness, and security (H2a, H2b, and H2c). As shown in Table 4, the indirect effect is considered significant when the 95% confidence interval does not include zero. The results indicate that collective psychological ownership mediates the relationships between community participation and residents’ sense of gain, happiness, and security. Therefore, H2a, H2b, and H2c are supported, further confirming the proposed model.

Finally, this study examined predictive effect indicators and predictive relevance. Community participation showed a predictive effect (f^2^) of 0.034 on collective psychological ownership, exceeding the threshold of 0.02. The R^2^ value was 0.382, exceeding the threshold of 0.19 (Chin, 1998). The predictive effects of community participation and collective psychological ownership on sense of gain ranged from 0.035 to 0.235, with an R^2^ of 0.353. Similarly, the predictive effects for happiness ranged from 0.062 to 0.154, with an R^2^ of 0.457. For security, the predictive effects ranged from 0.010 to 0.271, with an R^2^ of 0.316. Overall, these values generally met the minimum threshold requirements for f^2^ and R^2^. However, although community participation positively predicted residents’ sense of security, its effect size remained weak (f^2^ = 0.01). This suggests that, while community participation is positively related to residents’ sense of security, its unique incremental explanatory power for this outcome is limited. Rather, sense of security appears to be jointly shaped by individual background factors and psychological mechanism variables. To ensure model stability, cross-validation was performed, yielding Q^2^ values from 0.224 to 0.335, all above 0 and thus supporting the model’s predictive relevance (Henseler, 2010; Hair et al., 2019).

Overall, evaluation of the structural model revealed no multicollinearity problems. Finally, this study examined predictive effect indicators and predictive relevance (Table 5).

**Table 5:** R^2^, the effect size (f^2^) and the predictive relevance (Q^2^).

Criterion variable	Predictor variable	R^2^	f^2^	Q^2^
Collective psychological ownership	Community participation	0.382	0.034	0.287
Sense of gain	Community participation	0.353	0.035	0.246
	Collective psychological ownership		0.235	
Happiness	Community participation	0.457	0.062	0.335
	Collective psychological ownership		0.154	
Sense of security	Community participation	0.316	0.010	0.224
	Collective psychological ownership		0.271	

## 5 Conclusion and discussion

### 5.1 Conclusion

This study examines how community participation relates to rural tourism residents’ sense of gain, happiness, and security, and further tests collective psychological ownership as a mediating mechanism and government trust as a moderating condition. The results show, first, that community participation is positively associated with all three outcomes, with the strongest relationship for happiness, followed by sense of gain and sense of security. This is broadly consistent with prior studies showing that community participation contributes to residents’ positive perceptions (Lv and Xie, 2017; Chen and Zhang, 2022), while indicating that its effects vary across different perceptual dimensions. Second, collective psychological ownership significantly mediates the relationships between community participation and residents’ sense of gain, happiness, and security, with the strongest mediating effect observed for sense of security. This finding aligns with research showing that psychological ownership enhances positive attitudes (Fan et al., 2019; Ali et al., 2021), and suggests that collective ownership perception is an important mechanism in rural tourism destinations. Third, government trust strengthens the positive relationship between community participation and collective psychological ownership, supporting prior research that institutional trust shapes residents’ attitudes toward tourism development and governance arrangements (Ouyang et al., 2017; Kim et al., 2025). This further indicates that government trust is an important condition under which participation is translated into positive psychological outcomes.

### 5.2 Theoretical implications

First, by examining the relationships between community participation and residents’ sense of gain, happiness, and security, this study moves beyond prior research that has often focused on a single dimension and offers a more comprehensive analytical framework. These three dimensions are important measures of residents’ quality of life and are interrelated and mutually reinforcing (Zhang et al., 2022), yet existing research has largely examined them separately (Shang and Li, 2022). By analyzing how community participation relates to all three dimensions, this study addresses this limitation and enriches the literature on residents’ perceived quality of life.

Second, this study introduces collective psychological ownership into tourism research to explain how community participation predicts residents’ sense of gain, happiness, and security. Prior research on psychological ownership has mainly developed in organizational management and marketing and has focused primarily on the individual level, while studies on collective psychological ownership and its perceptual effects in tourism remain limited (Xu et al., 2022; Huo et al., 2023). By examining its mediating role in rural tourism settings, this study helps clarify the psychological mechanism linking community participation to residents’ positive perceptions and extends the theoretical application of collective psychological ownership.

Third, this study identifies government trust as a conditional factor linking community participation to residents’ collective psychological ownership. Although government trust has received extensive scholarly attention, residents’ trust in government remains underexamined in tourism research (Jia et al., 2021). By showing that government trust strengthens the positive relationship between community participation and collective psychological ownership, this study contributes to the literature on government trust and clarifies its moderating function in rural tourism settings.

### 5.3 Managerial implications

Grounded in community participation and focused on residents’ sense of gain, happiness, and security, this study offers practical guidance for improving quality of life in rural tourism destinations. Enhancing these dimensions among rural tourism residents is a key task in advancing comprehensive rural revitalization. The findings show that community participation is positively associated with all three dimensions, underscoring the need to strengthen resident engagement in rural tourism development. Specifically, destinations should support residents’ involvement in daily tourism management through training and resource provision, thereby enhancing their managerial competence and sense of responsibility (Lv and Xie, 2017). Regular consultation mechanisms can also be established around tourism planning, public space use, and festival organization, enabling residents to participate not only as labor providers but also as local stakeholders in village decision-making. In addition, fair and transparent benefit distribution is essential for increasing residents’ willingness to participate (Pang et al., 2024). Rural tourism destinations should ensure that development revenues are allocated clearly and that residents’ voices are incorporated into planning and policy formulation, especially on issues related to local governance, traditional architecture, and land-space development. Given the relatively weak unique effect of community participation on sense of security, destinations should also improve infrastructure and maintain public order to strengthen residents’ perceptions of village stability and the predictability of everyday life.

Second, community participation is positively linked with residents’ sense of gain, happiness, and security by fostering collective psychological ownership. Rural tourism destinations should therefore encourage residents to participate in community management and decision-making, grant them autonomy and voice, and strengthen their perceived control over the community (Xu et al., 2022). Education and training programs, such as festival preparation discussions and storytelling about village history and culture, can also enhance residents’ awareness of community tourism affairs and help them better understand village tourism operations, resource use, and internal collaboration (Jiang et al., 2024). Finally, residents should be encouraged to remain involved through cultural heritage transmission, public affairs maintenance, and community service, so that their long-term investment of time, emotion, and action strengthens their psychological bond with the village.

Third, government trust shapes how residents perceive their participation in community tourism development and functions as an important boundary condition for translating participation into positive psychological outcomes. It is therefore necessary to enhance rural tourism residents’ trust in government. Local governments should improve information disclosure, procedural transparency, and consultative responsiveness in key areas such as infrastructure construction, fund use, cultural resource utilization, and public affairs decision-making, enabling residents to understand policy bases, monitor implementation, and develop trust in governance fairness and development directions (Fang et al., 2022). During policy formulation and implementation, governments should also consider local community interests and avoid decisions that sacrifice community benefits in the name of development (Jia et al., 2021). Communication with communities should be strengthened through timely feedback and sustained follow-up to residents’ concerns, especially on sensitive issues such as ethnic cultural symbols, traditional architecture, and festival activities, where existing consultative orders and cultural authority structures should be respected. Finally, governments should increase tangible support for rural tourism through resource provision and policy guarantees, allowing residents to observe concrete efforts to advance local tourism and thereby strengthening their trust in government action.

### 5.4 Research limitations

Despite its contributions, this study has several limitations. First, the use of cross-sectional data limits causal inference; future research could employ longitudinal or experimental designs to further clarify the causal relationships examined here. Second, this study applies psychological ownership theory to explain how community participation predicts residents’ sense of gain, happiness, and security through possession psychology. Future research may draw on alternative frameworks, such as cultural identity or social capital, to further explain these pathways (Zheng et al., 2022). Third, although the study selected five rural tourism destinations based on life-cycle stages, these sites may not represent all rural tourism contexts. Given cultural diversity and regional specificity, future studies should examine different geographical and cultural settings to test the generalizability and robustness of the proposed model. Finally, while this study demonstrates the moderating role of government trust, other public organizations also matter in rural tourism development. Future research could therefore examine how residents’ trust in other institutions influences the formation of collective psychological ownership in community tourism (Jia et al., 2021).

## Data Availability

The original contributions presented in the study are included in the article/supplementary material, further inquiries can be directed to the corresponding author.
